# Evaluating post-concussion symptom profiles using the convergence insufficiency symptom survey in a pediatric and adolescent cohort

**DOI:** 10.3389/fnins.2026.1799528

**Published:** 2026-03-25

**Authors:** Debora Ghosh, Sophia Marusic, Jennifer X. Haensel, Carissa H. Wu, Kristin E. Slinger, Neerali Vyas, Christabel A. Ameyaw Baah, Amber Hu, Joellen Leonen, Caitlyn Y. Lew, Gayathri Srinivasan, Amir Norouzpour, Erin Jenewein, Siva Meiyeppen, Mitchell Scheiman, Tawna L. Roberts, Aparna Raghuram

**Affiliations:** 1 Boston Children's Hospital, Harvard Medical School, Boston, MA, United States; 2 Spencer Center for Vision Research, Byers Eye Institute at Stanford University, Palo Alto, CA, United States; 3 Pennsylvania College of Optometry at Salus University, Philadelphia, PA, United States

**Keywords:** accommodation, adolescent, concussion, vergence, vision symptoms, pediatric

## Abstract

**Purpose:**

This multicenter study aimed to compare symptoms in pediatric and adolescent patients with and without concussion using the Convergence Insufficiency Symptom Survey (CISS). We further examined symptom profiles of concussed patients with and without vergence and/or accommodation deficits.

**Methods:**

Children aged 8 to <18 years, diagnosed with concussion 4 weeks to 12 months prior and visually normal controls underwent comprehensive testing of vergence and accommodation. Participants completed the 15-item CISS, with somatic (CISS-S), performance (CISS-P), and vision (CISS-V) subscores. Mann–Whitney U tests compared total CISS scores and normalized subscores between concussed and control groups, and concussed participants with and without vergence and/or accommodative deficits.

**Results:**

Among 66 eligible participants (34 concussed, median age 15.0 [interquartile range, IQR: 12.2–16.0]; 32 controls, median age 13.0 [11.0–14.0]), concussed individuals had substantially higher total CISS scores (median 26.0 [19.25–36.75] vs. 4.0 [1.0–7.0]; *p* < 0.0001) and higher CISS-S, CISS-P and CISS-V subscores than controls (all *p* < 0.001). Within the concussion group, 76.5% (26 of 34) demonstrated vergence and/or accommodative deficits, showing significantly higher total CISS scores (31.50 [22.25–38.75] vs. 19.50 [15.75–21.25]; *p* = 0.022), higher normalized CISS-V subscores (1.33 [0.75–2.25] vs. 0.50 [0.00–0.67]; *p* = 0.005) and CISS-P subscores [2.50 [2.05–3.20] vs. 1.50 [1.15–2.10]; *p* = 0.047] compared to those without such deficits. No significant difference in CISS-S (2.14 [1.61–2.82] vs. 1.43 [0.82–1.75], *p* = 0.084) was observed between concussed groups.

**Conclusion:**

Vergence and accommodation deficits were associated with higher CISS vision and performance related subscores. Elevated symptom reporting in the somatic and performance subscores in concussed may indicate strain in the vergence and accommodative system, as evidenced by the increased subjective discomfort and difficulty during tasks like reading and near work. Concurrent assessment of vergence and accommodation alongside CISS symptom subscores may identify patients for vision rehabilitation, aimed at improving vergence and accommodation function and reducing somatic and performance-related symptoms.

## Introduction

Concussion, a form of mild traumatic brain injury (mTBI), is a significant and growing public health concern in the pediatric population. In 2022, approximately 2.3 million children and adolescents (3.2%) in the United States were diagnosed with a concussion or brain injury, with prevalence increasing with age: from 1.0% in children aged 0–5 years, to 2.3% in those 6–11 years, and 5.9% in adolescents aged 12–17 years (Elgaddal and Black, 2023). National surveys report lifetime concussion/head injury estimates in youth ranging from 2.5 to 18.3%, with the highest prevalence found in adolescents aged 13–17 years (Haarbauer-Krupa et al., 2021). Annually, over 120,000 emergency department visits occur in children under 18 due to mTBI, particularly in males (Singichetti et al., 2018). Approximately 87% of pediatric TBIs recorded in national trauma databases are classified as mild, and most children recover within a few weeks (Yue et al., 2016). However, a subset of children experience prolonged symptoms, hospitalization, or complications—demonstrating that even mild TBIs can lead to an extended recovery depending on injury severity, risk factors, and mechanism of injury (Yue et al., 2016).

After concussion, many children and adolescents experience a range of symptoms across physical, cognitive, emotional, and sleep related systems (Harmon et al., 2013). Given that processing of visual information is supported by extensive cortical circuitry, the visual system is particularly vulnerable post-concussion, making visual symptoms highly prevalent (Ventura et al., 2015; Wu et al., 2018). Commonly reported symptoms include double vision, blurred vision, headaches during near work, light sensitivity, and difficulty reading or focusing (Ventura et al., 2015; Gallaway et al., 2017; Wiecek et al., 2021; Schmitz et al., 2023). Studies consistently show that convergence insufficiency (CI) and accommodative dysfunction are among the most common diagnoses post-concussion (Gallaway et al., 2017; Gowrisankaran et al., 2021; Scheiman et al., 2021). Convergence insufficiency is a binocular vision-related disorder in which the eyes do not work together effectively during near tasks, resulting in difficulty maintaining alignment and fusion (Scheiman et al., 2020). Accommodative dysfunction is characterized by an impaired ability to focus and/or sustain focus during near visual activities (Daum, 1983; Scheiman et al., 2011).

In our recent study, we reported that these vision-related dysfunctions may persist for months post-injury and are associated with prolonged recovery and reduced quality of life (Marusic et al., 2024). Frequency of vergence or accommodative dysfunction in both the subacute (15 days–12 weeks) and chronic (>12 weeks–1 year) phases from the time since concussion were similar, indicating that such deficits may not resolve spontaneously and could benefit from evaluation by a specialized eye care provider (Marusic et al., 2024).

The Convergence Insufficiency Symptom Survey (CISS) is a 15-item questionnaire developed to assess symptoms in patients with CI (Scheiman, 2008). While initially designed and validated to diagnose symptomatic CI and used as an outcome measure in large clinical treatment trials like the CITT and CITT-Attention and Reading Trial (CITT-ART Investigator Group, 2019; Scheiman, 2008, 2015; Rouse et al., 2009), the CISS has since been adapted for use in concussion populations to capture symptom burden (Gowrisankaran et al., 2021) when doing near activities. The CITT established the clinical utility of the CISS in children aged 9–17 years, demonstrating the effectiveness of vergence/accommodative therapy in reducing symptoms (Scheiman, 2008) in symptomatic children with CI. Standard concussion scales such as the Post-Concussion Symptom Scale (PCSS) only assess whether a patient declares they have “Sensitivity to Light,” or have “vision problems” which does not capture the variety of vision-related symptoms that may present following concussion (Gowrisankaran et al., 2021). Although not a diagnostic tool for identifying CI following concussion, the CISS helps clinicians identify symptomatic individuals who may benefit from a comprehensive visual function assessment (Gowrisankaran et al., 2021; Vyas et al., 2025).

Our earlier retrospective study has shown that the CISS is sensitive to the broader symptomatology associated with post-concussion vergence and/or accommodation deficits and may offer a cost-effective, accessible tool for tracking symptoms outside of eye care clinics (Gowrisankaran et al., 2021). Additionally, studies suggest that dividing the CISS into subscores itemized into different domains such as somatic (e.g., headaches, eye strain), performance (e.g., re-reading lines, poor concentration), and vision-related (e.g., double vision, blurring) can help characterize symptom profiles following naturally occurring CI (Barnhardt et al., 2012), dyslexia (Raghuram et al., 2019b), and concussion (Vyas et al., 2025).

Using the CISS, we have demonstrated that individuals who have experienced a concussion report significantly higher somatic-related subscores in comparison to visually normal controls. Additionally, our research indicates that vergence and/or accommodation deficits following a concussion were associated with higher total CISS scores and vision-related subscores in a pediatric and adolescent cohort (Vyas et al., 2025). However, the previous study was retrospective and used a control group from a different study; in the current study, we aim to address these limitations to strengthen clinical recommendations for post-concussion care.

In this paper, we present a prospective study designed to compare vergence and/or accommodation deficits and symptom burden in children and adolescents with and without a history of concussion using the CISS. Our objectives are twofold: first, to evaluate total CISS scores and subscores across somatic-, performance-, and vision-related subscores between concussed and non-concussed participants; and second, to determine whether symptom severity as measured by the CISS differs between concussed individuals with and without identified vergence and/or accommodation deficits. We hypothesized that concussed participants would report significantly greater overall symptom burden than non-concussed controls, and within the concussion group, those with vergence and/or accommodation deficits would exhibit higher CISS subscores, particularly in vision-related subscores.

## Methods

### Institutional review board approval

This study received approval from the Institutional Review Boards of Boston Children’s Hospital (Boston, Massachusetts, United States), Stanford University (Stanford, California, United States), and Salus University (Elkins Park, Pennsylvania, United States). Written informed consent was obtained from a parent or legal guardian of each participant, and written assent was obtained from all participants prior to enrolment in the study.

### Participant recruitment and eligibility

Participants were recruited from Boston Children’s Hospital, Stanford University, and the Pennsylvania College of Optometry at Salus University. Control participants were enrolled through word-of-mouth referrals, departmental staff, local community, and patients attending routine eye examinations at the affiliated clinics. Participants with concussion were recruited from dedicated concussion clinics at Boston Children’s Hospital and Stanford University or were referred to the vision care practices of the senior investigators (TLR and AR) for post-concussion assessment. These referrals were made for individuals experiencing persistent post-concussion symptoms, regardless of vision-specific complaints.

The inclusion criteria for the concussion group required a physician-diagnosed concussion in accordance with the Berlin Consensus Statement on Concussion in Sport, with the study visit occurring 4 weeks to 12 months post-injury. Control participants were eligible if they had no history of concussion and no known accommodative or vergence deficits. All participants were aged between 8 and <18 years, demonstrated best-corrected distance visual acuity of 20/25 or better in each eye, and wore appropriate refractive correction. Detailed inclusion and exclusion criteria are provided in Table 1 and Supplementary methods 1.

**Table 1:** Inclusion and exclusion criteria.

Inclusion criteria	Exclusion criteria
Age 8–18 years Best-corrected visual acuity of 20/25 or better in each eye Appropriate refractive correction worn Concussed group: Diagnosed with a concussion by treating physician per Berlin Consensus Statement Time since injury between 4 weeks and 12 months Control group: No prior history of concussion No known vergence or accommodation deficits	History of amblyopia History of strabismus or diplopia Prior in-office vision therapy Ocular trauma affecting visual or oculomotor function Structural abnormalities of the cornea, lens, or central retina Constant/intermittent esotropia at distance or near Constant exotropia at near or distance Vertical heterophoria ≥2 prism diopters (Δ) at distance or near Manifest or latent nystagmus Neurological or ocular conditions affecting vergence, accommodation, or eye movements Inability to reliably perform study-related vision assessments

### Symptom assessments

#### Convergence insufficiency symptom survey

The CISS is a 15-item questionnaire (Table 2) validated for use in patients with CI (Scheiman, 2008). Each item is rated on a Likert scale ranging from “never” (0) to “always” (4), yielding a total score between 0 and 60, with higher scores indicating greater symptom severity. The CISS items were further divided into subcategories: 7 somatic-related symptoms (CISS-S), 5 performance-related symptoms (CISS-P), and 3 vision-related symptoms (CISS-V) (Scheiman, 2008; Barnhardt et al., 2012; Vyas et al., 2025). CISS subscores were normalized by dividing the category’s total response score by the number of items in the category to control for each subscore containing different numbers of items (Vyas et al., 2025).

**Table 2:** Convergence insufficiency symptom survey (CISS).

Symptom	Never	Not very often	Some-times	Fairly often	Always
1. Do your eyes feel tired when reading or doing close work?	0	1	2	3	4
2. Do your eyes feel uncomfortable when reading or doing close work?	0	1	2	3	4
3. Do you have headaches when reading or doing close work?	0	1	2	3	4
4. Do you feel sleepy when reading or doing close work?	0	1	2	3	4
5. Do you lose concentration when reading or doing close work?	0	1	2	3	4
6. Do you have trouble remembering what you have read?	0	1	2	3	4
7. Do you have double vision when reading or doing close work?	0	1	2	3	4
8. Do you see the words move, jump, swim or appear to float on the page when reading or doing close work?	0	1	2	3	4
9. Do you feel like you read slowly?	0	1	2	3	4
10. Do your eyes ever hurt when reading or doing close work?	0	1	2	3	4
11. Do your eyes ever feel sore when reading or doing close work?	0	1	2	3	4
12. Do you feel a “pulling” feeling around your eyes when reading or doing close work?	0	1	2	3	4
13. Do you notice the words blurring or coming in and out of focus when reading or doing close work?	0	1	2	3	4
14. Do you lose your place while reading or doing close work?	0	1	2	3	4
15. Do you have to re-read the same line of words when reading?	0	1	2	3	4

Somatic-related symptoms (CISS-S) are shaded in blue, performance-related symptoms (CISS-P) in pink, vision-related symptoms (CISS-V) in yellow.

### Vision and visual function assessment

Following the administration of the CISS, each participant underwent a comprehensive visual function examination conducted by a pediatric optometrist, including assessment of distance and near visual acuity, stereopsis, ocular motility, ocular alignment, vergence, and accommodation. Ocular alignment was evaluated using the cover-uncover test, with deviation magnitude measured by the prism and alternate cover test. The vergence assessment included near-point of convergence (NPC), near vergence facility, and fusional amplitudes for near convergence and divergence. Accommodation was evaluated using amplitude of accommodation (AA) and monocular accommodative facility. Any examiner- or participant-noted difficulties with base-out (convergence demand) or base-in (divergence demand) prisms during vergence facility testing were noted. Similarly, difficulties with minus (increased demand) or plus (decreased demand) lenses during accommodative facility testing were noted. Diagnoses related to vergence and accommodation were made based on visual function testing results and criteria described in Table 3 (Raghuram et al., 2019a; Gowrisankaran et al., 2021; Wu et al., 2025). Further details of the examination procedures can be found in Supplementary methods 1.

**Table 3:** Vergence and accommodation diagnostic criteria.

Clinical diagnosis and findings	Criteria
Vergence diagnoses	
**Convergence insufficiency** Near deviation Near point of convergence Convergence fusional amplitudes at near Vergence facility (3ΔBI/12ΔBO)	*First criteria and one other must be met* ≥4 Δ near exophoria more than distance >6 cm ≤15 Δ blur/break^a^ or failing Sheard’s criterion^b^ ≤9 cpm, BO prism harder
**Convergence deficit** Near point of convergence Convergence fusional amplitudes at near Vergence facility (3ΔBI/12ΔBO)	*First criteria and one other must be met* >6 cm ≤15 Δ blur/break^a^ or failing Sheard’s criterion^b^ ≤9 cpm, BO prism harder
**Convergence excess** Near Deviation Divergence fusional amplitudes at near Vergence facility (3ΔBI/12ΔBO)	*First criteria and one other must be met* ≥3 Δ esophoria at near ≤8 Δ blur/break^a^ or failing Sheard’s criterion^b^ ≤9 cpm, BI prism harder
**Divergence deficit** Divergence fusional amplitudes at near Vergence facility (3ΔBI/12ΔBO)	*Must meet both criteria* ≤8 Δ blur/break^a^ or failing Sheard’s criterion^b^ ≤9 cpm, BI prism harder
**Fusional vergence dysfunction** Convergence fusional amplitudes at near Divergence fusional amplitudes at near Vergence facility (3ΔBI/12ΔBO)	*Must meet first two criteria OR fail third* ≤15 Δ blur/break^a^ or failing Sheard’s criterion^b^ ≤8 Δ blur/break^a^ or failing Sheard’s criterion^b^ ≤9 cpm, BI and BO prisms both difficult
Accommodative diagnoses	
**Accommodative insufficiency** Accommodative amplitude Accommodative facility (±2D)	*Must meet one criteria* <11 D ≤6 cpm, (−) lens difficult
**Accommodative excess** Accommodative Amplitude Accommodative Facility (±2D)	*Must meet both criteria* ≥11 D (normal) ≤6 cpm, (+) lens difficult
**Accommodative infacility** Accommodative infacility (±2D)	*Must meet criteria* ≤6 cpm, (+) and (−) lens equally difficult
**Accommodative dysfunction** Accommodative Amplitude Accommodative Facility (±2D)	*Most meet both criteria* <11 D ≤6 cpm, (+) lens difficult
**Accommodative insufficiency and accommodative infacility** Accommodative Amplitude Accommodative Facility (±2D)	*Must meet both criteria* <11 D ≤ 6 cpm, (+) and (−) lens equally difficult

BI, base-in prism; BO, base-out prism; cpm, cycles per minute; D, diopter; Δ, prism diopter.

a Blur/break refers to the point at which the participant reports that the target is blurry, or if there is no blur, when it splits into two.

b Sheard’s Criterion: Compensating vergence range (positive or negative fusional vergence) of at least two times near heterophoria.

### Statistical analysis

Categorical data were summarized as proportions and frequencies, while continuous data were presented as medians and interquartile ranges (IQR). Since a Shapiro–Wilk test indicated non-normality for CISS scores, non-parametric Mann–Whitney U tests were conducted to compare total CISS scores as well as subscores between concussed and control participants. Separately for the control and concussed groups, Friedman tests were used to compare normalized subscores within each group. A two-sided alpha-value of 0.05 was set as the threshold for statistical significance in all analyses. Wilcoxon signed rank post-hoc tests were used for any pairwise comparisons between normalized subscores within each group, with an adjusted alpha-value of 0.017. Finally, Mann Whitney U tests were conducted to compare total CISS scores and subscores between concussed participants with and without vergence and/or accommodative deficits. To assess symptom severity scores in the concussed and control groups, the mean scores across participants for individual CISS items were calculated. All statistical tests were conducted using RStudio (version 4.4.1).

## Results

Of the 71 participants recruited, 66 met inclusion criteria. Participants were excluded due to ineligible visual acuity (*n* = 3), reports of constant diplopia (*n* = 1), and esotropia (*n* = 1). Among the 66 remaining participants, 34 were concussed (25 female, 9 male; median age 15.0 years [IQR: 12.2 to 16.0]) and 32 were controls (18 female, 14 male; median age 13.0 years [IQR: 11.0 to 14.0]). All consecutive control participants recruited for the study were enrolled. No subjects were excluded based on binocular vision findings. Only one participant was excluded from the control group because of esotropia. The median time since concussion was 107.0 days (IQR: 80 to 118), with 24 participants (71%) reporting a sport-related concussion, 9 (26%) indicating other causes such as falls, and 1 (3%) being due to a motor vehicle accident. Participants reported having sustained 1, 2, or more than 3 concussions in 52, 35, and 13% cases, respectively.

### CISS scores between concussed and control group

**Figure 1:**
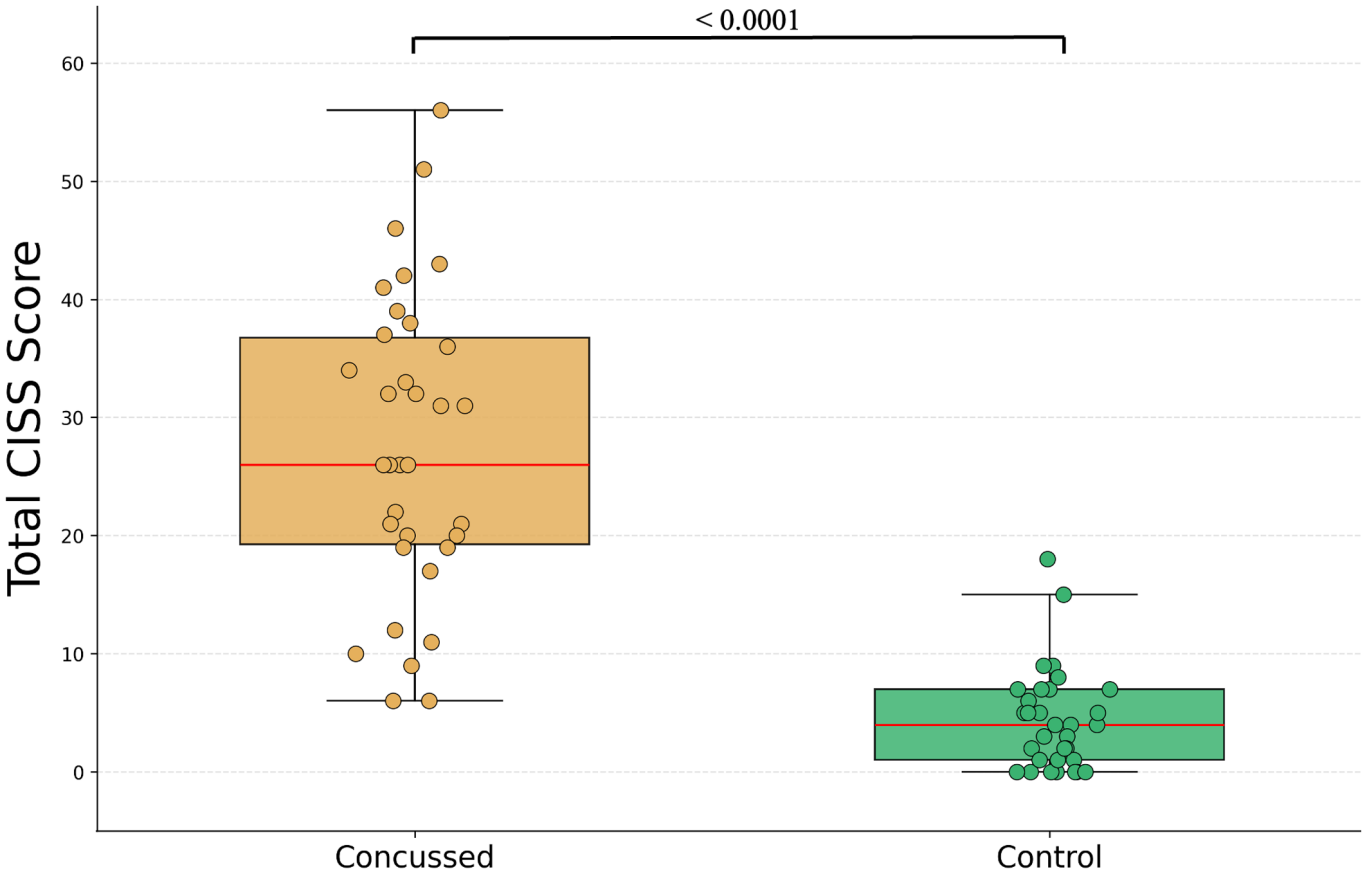
Total CISS score in concussed and control participants. Box plot showing the distribution of total Convergence Insufficiency Symptom Survey (CISS) scores in concussed (orange) and control (green) participants. Individual data points are overlaid as semi-transparent circles. Boxes represent the interquartile range (IQR), red center lines indicate the median, and whiskers denote the minimum and maximum values.

**Figure 2:**
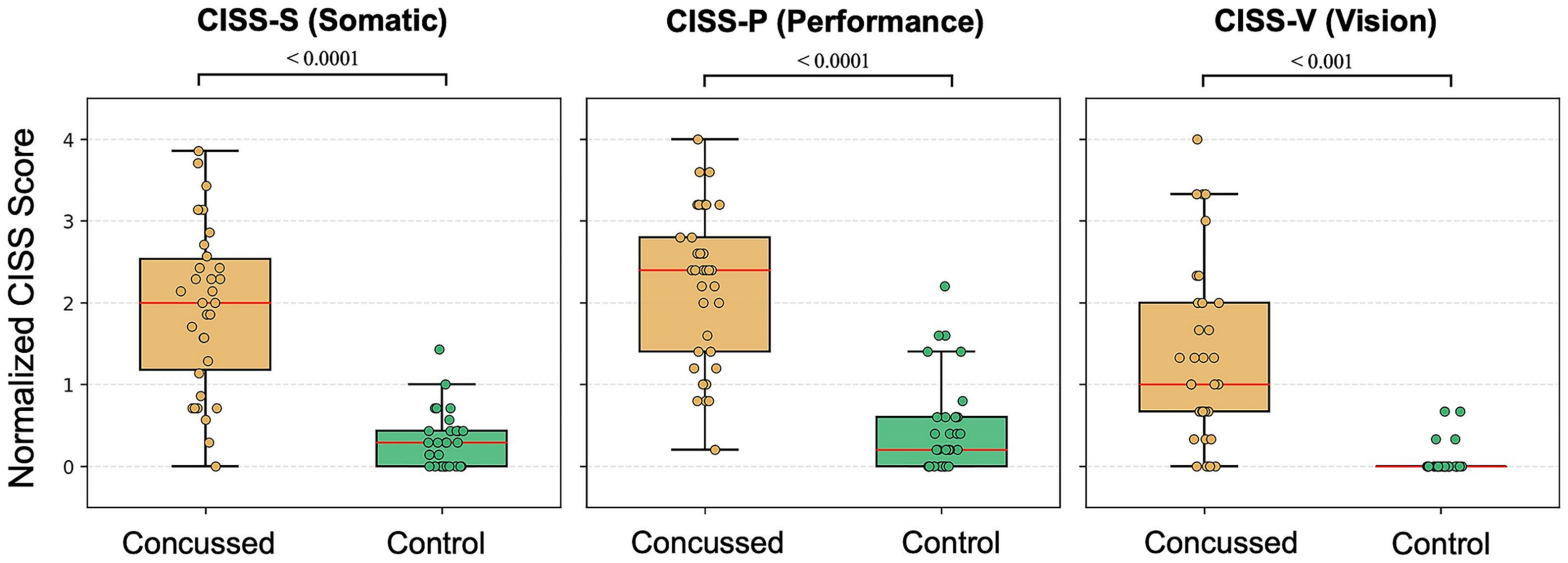
Normalized CISS subscores in concussed and control groups. Box plots illustrating normalized CISS subscores for somatic (CISS-S), performance (CISS-P), and vision (CISS-V) domains in concussed (orange) and control (green) participants. Individual participant data are shown as semi-transparent circles. Boxes indicate the interquartile range, red center lines represent the median, and whiskers denote the minimum and maximum values.

The Mann–Whitney U test showed that the median total CISS score [IQR] was significantly higher in concussed participants (26.0 [19.25 to 36.75]) than in controls (4.0 [1.0 to 7.0]; U = 1059.0, *p* < 0.0001; Figure 1). Compared with controls, concussed participants also had significantly higher normalized median subscores for CISS-S (concussed: 2.00 [(1.18 to 2.54)], controls: 0.29 [0.00 to 0.43]; U = 1023.1, *p* < 0.0001), CISS-P (concussed: 2.40 [1.40 to 2.80], controls: 0.20 [0.0 to 0.60]; U = 1020.5, *p* < 0.0001), and CISS-V (concussed: 1.00 [0.67 to 2.00], controls: 0.00 [0.00 to 0.00]; U = 983.0, *p* < 0.001; Figure 2).

CISS-P subscores were highest in both the concussed and control group, followed by CISS-S and CISS-V subscores. The Friedman test showed significant differences among the subscores in the concussed group [χ^2^(2) = 23.93, *p* < 0.001] as well as the control group [χ^2^(2) = 14.67, *p* < 0.001]. For the concussed group, Wilcoxon signed rank post-hoc tests indicated significantly higher scores for CISS-P (2.40 [1.40 to 2.80]) than CISS-V (1.00 [0.67 to 2.00]; z = −4.20, *p* < 0.001) and CISS-S (2.00 [1.18 to 2.54]) than CISS-V (1.00 [0.67 to 2.00]; z = −3.56, *p* < 0.001) but no significant difference between CISS-P (2.40 [1.40 to 2.80]) and CISS-S (2.00 [1.18 to 2.54]; z = −1.93, *p* = 0.054). In the control group the trend was similar, with scores for CISS-P (0.20 [0.0 to 0.60]) significantly higher than CISS-V (0.00 [0.00 to 0.00]; z = −3.19, *p* < 0.001) and CISS-S (0.29 [0.00 to 0.43]) than CISS-V (0.00 [0.00 to 0.00]; z = −3.18, *p* < 0.002). However, no significant difference was observed between CISS-P (0.20 [0.0 to 0.60]) and CISS-S (0.29 [0.00 to 0.43]; z = −1.24, *p* = 0.215) subscores.

### CISS scores within concussed group

**Figure 3:**
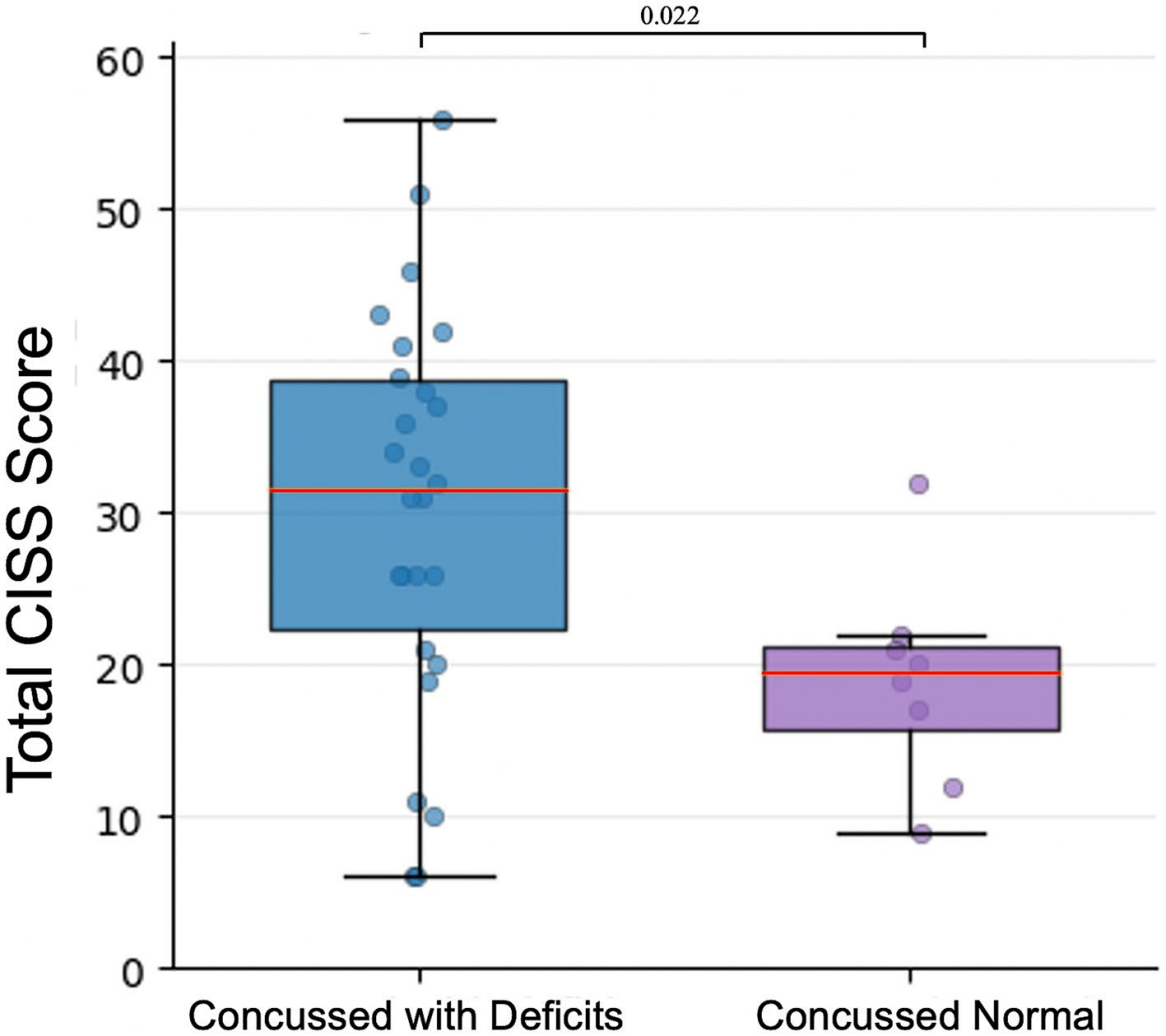
Total CISS score in concussed participants with and without vergence and/or accommodation deficits. Box plot comparing total CISS scores between concussed participants with vergence and/or accommodation deficits (Concussed with Deficits; blue) and concussed participants without vergence or accommodation deficits (Concussed Normal; purple). Individual data points are overlaid. Boxes represent the interquartile range, the red center lines show median values, and whiskers indicate the minimum and maximum scores.

**Figure 4:**
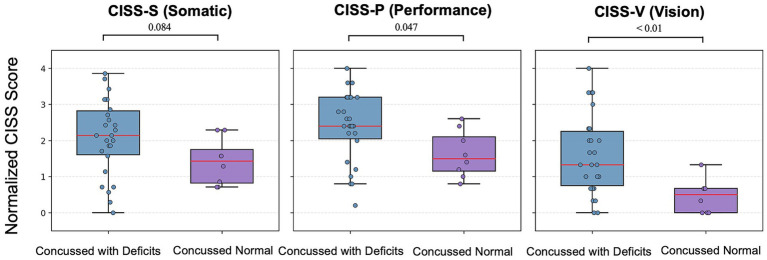
Normalized CISS subscores in concussed participants with and without vergence and/or accommodation deficits. Box plots showing normalized CISS subscores for somatic (CISS-S), performance (CISS-P), and vision (CISS-V) symptoms in concussed participants with vergence and/or accommodation deficits (blue) compared with concussed participants without vergence and/or accommodation deficits (purple). Individual data points are displayed as semi-transparent circles. Boxes represent the interquartile range, red center lines indicate the median, and whiskers denote the minimum and maximum values.

Among the 34 concussed participants, 26 (76.5%) had vergence and/or accommodation deficits. Twenty one (80.8%) had both vergence and accommodation deficits; 5 (19.2%) had only accommodation deficits; none of the participants had only vergence deficits (Wu et al., 2025; Tables 4, 5). The median total CISS scores were significantly higher in the concussed group with vergence and/or accommodation deficits (31.50 [22.25 to 38.75]) compared to concussed participants without deficits (19.50 [15.75 to 21.25]; U = 47, *p* = 0.022; Figure 3). Compared with participants without vergence and/or accommodation deficits, those with deficits also had significantly higher normalized median subscores for CISS-V (concussed with deficits: 1.33 [0.75 to 2.25], concussed without deficits: 0.50 [0.00 to 0.67]; U = 35, *p* < 0.01) and for CISS-P (concussed with deficits: 2.40 [2.05 to 3.20], concussed without deficits: 1.50 [1.15 to 2.10]; U = 54.5; *p* = 0.047). No significant group difference was observed for CISS-S (concussed with deficits: 2.14 [1.61 to 2.82], concussed without deficits 1.43 [0.82 to 1.75]; U = 61, *p* = 0.084; Figure 4).

**Table 4:** Demographic and clinical findings of concussed participants with and without vergence and/or accommodation deficits.

Group	Concussed with deficits	Concussed without Deficits
N	26	8
Mean age (years) [IQR]	15 [4]	15.5 [3.5]
Sex (% female)	21 (80.8%)	4 (50%)
Median number of concussion [IQR]	1 [1]	1 [1]
Median days since concussion [IQR]	107 [41]	103 [38.5]
Median NPC Break [IQR] (cm)	10.0 [6.7]	5.42 [1.9]
Median Break Positive Fusional Vergence at Near (Base Out) [IQR] (Δ)	15.0 [15.3]	20.7 [9.0]
Median Break Negative Fusional Vergence at Near (Base In) [IQR] (Δ)	14.0 [2]	14.0 [2]
Median vergence facility [IQR] (cpm)	9.75 [7]	11.0 [4.5]
Median accommodative amplitude [IQR] (D)	8.4 [2.9]	11.7 [1.6]

**Table 5:** Descriptive CISS-subscores data for concussed participants with and without vergence and/or accommodation deficits.

Median CISS score [IQR]	Concussed with deficits (26)	Concussed without deficits (8)	Mann–Whitney U statistic	*p*-value
Total CISS Score	31.50 [16.5]	19.50 [5.5]	47	0.022
Normalized Median CISS-V Subscore	1.33 [1.5]	0.50 [0.67]	35	< 0.01
Normalized Median CISS-P Subscore	2.40 [1.15]	1.50 [0.95]	54.5	0.047
Nomalized Median CISS-S Subscore	2.14 [1.21]	1.43 [0.93]	61	0.084

### Symptom severity in control and concussed groups

**Figure 5:**
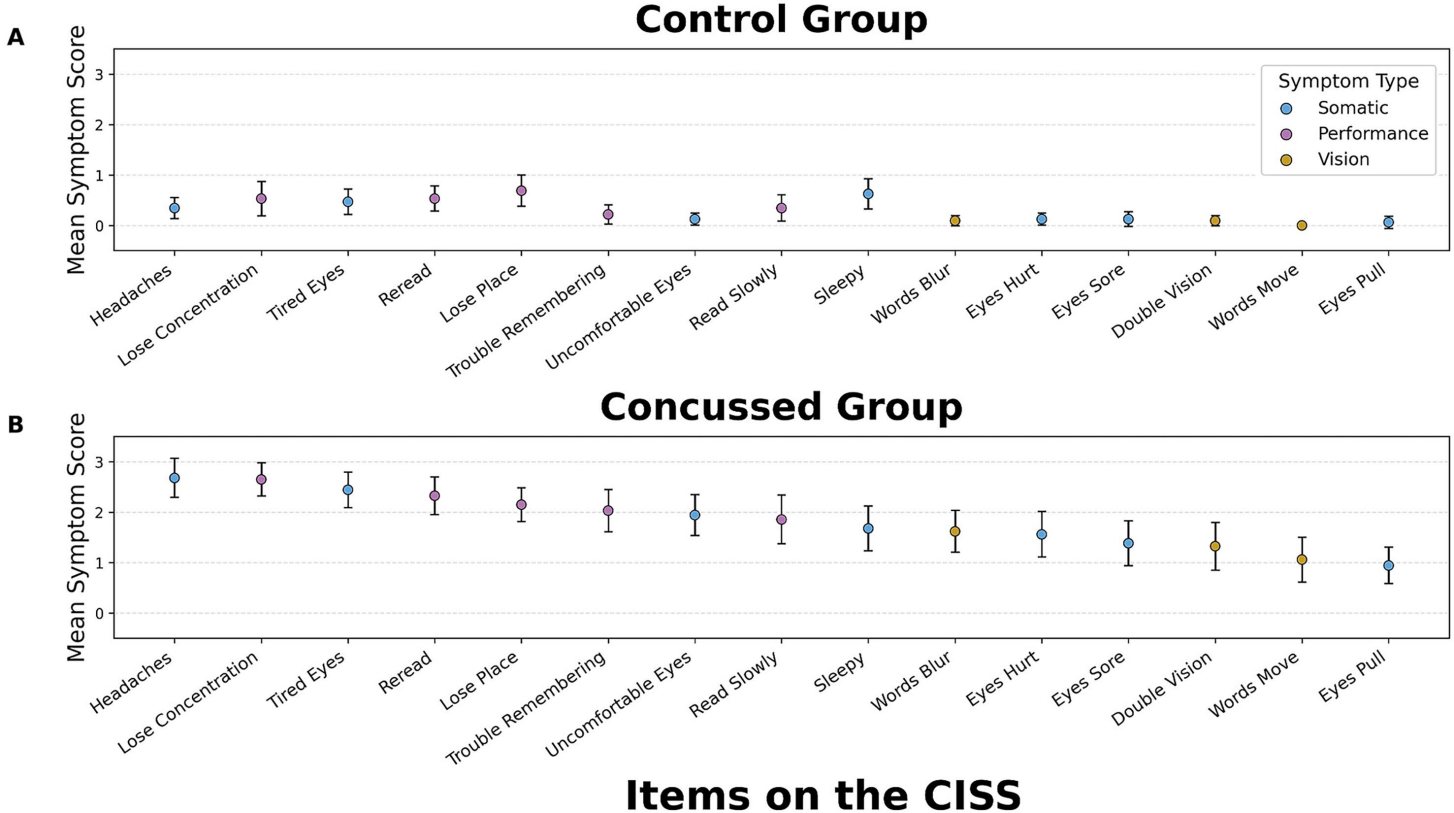
Interval plot of CISS symptom severity for control and concussed participants (95% CI). Interval plots showing mean symptom severity across individual items of the convergence insufficiency symptom survey (CISS) in control and concussed participants (Individual items listed in Table 2). Symbols represent the mean symptom severity for each CISS item, and vertical error bars indicate 95% confidence intervals. Symptoms are ordered from highest to lowest mean severity for the concussed group. Items are color-coded by symptom domain: somatic (blue), performance (pink), and vision (yellow).

Symptom severity across the 15 CISS items revealed distinct differences in symptom reporting trends between control and concussed participants. In the control group, symptom severity was generally low, with most symptoms receiving a mean score close to zero. The top five reported symptoms in this group were “Lose Place,” “Sleepy,” “Lose Concentration, “Re-Read,” and “Tired Eyes,” with mean symptom scores ranging from 0.2 to 0.75. CISS-V items such as “Double Vision” and “Words Blur” were among the least reported in this group, with mean scores closer to zero (Figure 5A).

In contrast, the concussed group demonstrated a greater symptom burden across all categories. The distribution of symptom burden was similar between those with vergence and/or accommodation deficits and those without, with only slight differences in magnitude; therefore, these subgroups were combined and analyzed as a single concussed group. The most frequently reported symptoms were “Headaches,” “Lose Concentration,” “Tired Eyes,” “Re-Read,” and “Lose Place,” with mean symptom scores ranging from 0.6 to 2.8. Headaches had the highest mean symptom severity score. CISS-V items, such as “Words Blur” and “Double Vision,” were reported more frequently in the concussed group, particularly among participants with vergence and/or accommodation deficits, compared to controls (Figure 5B).

## Discussion

Concussed participants reported significantly higher total CISS scores than visually normal controls with no history of concussion, along with higher scores on somatic, performance, and vision-related subscores. Within the concussed cohort, those with vergence and/or accommodative deficits experienced greater symptom severity across all CISS subscores, particularly for CISS-V and CISS-P, compared to concussed participants without such deficits. Accommodative and vergence deficits were common in our concussed cohort, consistent with previous studies (Master et al., 2016; Gallaway et al., 2017; Wiecek et al., 2021). Symptom reporting can vary depending on how visual complaints are perceived or described by patients; for example, visual discomfort might manifest as general fatigue, headaches, losing concentration, or reading difficulties, which can fall under general somatic- or cognitive-related symptoms without specifically recognized as stemming from visual function problems (Danielli et al., 2023; Leinonen et al., 2024). This emphasizes using the CISS to characterize symptoms following concussion and using the subscores to further help understand the presence of vergence and/or accommodation deficits or symptom provocation when doing near work (Trbovich et al., 2019; Vyas et al., 2025). The CISS can help identify symptom patterns and guide referral decisions even when overt vision symptoms are not the primary complaint (Master et al., 2016; Trbovich et al., 2019).

Interestingly, both concussed and control groups demonstrated the highest symptom burden in CISS-P items, although the magnitude was significantly greater in the concussed group. This trend mirrors findings in patients with naturally occurring CI. In the CITT randomized clinical trial, performance-related symptoms were more frequently endorsed than eye-related symptoms among children with symptomatic naturally occurring CI (Scheiman, 2008; Barnhardt et al., 2012). In our study, the similar CISS-P and CISS-S scores, combined with elevated CISS-V scores in the concussed group, suggest a broader neurological disruption affecting multiple systems, rather than just vision-related dysfunction.

CISS-S in our concussed cohort were reported with similar frequency as CISS-P subscores. This pronounced somatic symptom burden may reflect underlying discomfort when facing visually demanding tasks. Notably, even in the absence of measurable vergence or accommodation deficits, elevated somatic and performance symptom reporting may suggest subjective experience of discomfort and difficulty with visual system use. Therefore, patients with normal vergence and accommodation findings who report high somatic and performance symptoms might still experience ongoing subjective discomfort and strain. Rehabilitation strategies focused on alleviating symptoms even when clinical findings are normal could effectively manage persistent post-concussion symptoms. These findings underscore the importance of systematically evaluating visual function in concussed patients, as addressing visual function related deficits can inform targeted interventions to help reduce symptom burden and facilitate recovery (Gowrisankaran et al., 2021; Vyas et al., 2025).

The distribution of symptoms across subscores in concussed patients may indicate disruption in the neural systems beyond the oculomotor circuit. The visual system recruits an extensive cortical network, and post-concussive vulnerability may extend to those networks that also support visual attention, memory, and visual-motor coordination (Shah-Basak et al., 2018; Sangoi et al., 2025). These mechanisms might explain somatic symptoms such as headaches or tired eyes during reading or close work, even in the absence of measurable vergence deficits.

A large proportion of our concussed participants were in the chronic phase of recovery (median time since injury: 107 days), yet symptom burden remained high. This supports previous findings that while vergence and accommodative deficits may persist months post-injury, symptom intensity can also remain high regardless of time since injury (Leinonen et al., 2024; Marusic et al., 2024; Fenner et al., 2025). Furthermore, the high prevalence of multiple concussions in our sample (48.4% with more than one concussion) may also contribute to greater symptom severity and complexity, as cumulative injuries have been associated with prolonged recovery and increased risk of vergence and/or accommodation deficits (Master et al., 2016). Studies suggest that persistent vergence and/or accommodation deficits post-concussion often require intervention and do not resolve spontaneously, reinforcing the need for early identification and monitoring (Marusic et al., 2024).

This study reinforces the utility of the CISS in identifying patients with potential vision-related deficits following concussion. Notably, when the CISS-V subscores are flagged as concerning, they can help pinpoint children and adolescents with vergence and/or accommodation deficits. This correlation was observed in our retrospective study (Vyas et al., 2025) and was corroborated with this prospective study. Although not a diagnostic tool, the CISS can identify individuals with pronounced symptoms who may benefit from comprehensive visual function assessment and, when appropriate, vision rehabilitation (Raghuram et al., 2019a; Trbovich et al., 2019; Gowrisankaran et al., 2021; Scheiman et al., 2021; Vyas et al., 2025). Prior research indicated that patients with post-concussion convergence and accommodative insufficiencies have a favorable response to vision rehabilitation, with 85% achieving either symptom resolution or improvement (Gallaway et al., 2017). However, a recent scoping review highlights a critical need for high-quality randomized clinical trials to thoroughly evaluate the effectiveness of vison rehabilitation intervention following concussion (Alvarez et al., 2025; Chen et al., 2025).

This study has several limitations. First, the number of concussed participants without vergence and accommodation deficits was relatively low, limiting subgroup comparisons and necessitating a larger sample size to confirm the replicability of these findings. Second, the characterization of mTBI may have been heterogenous, as mechanisms of injury and impact characteristics varied across participants. The modest sample size also limited our ability to include age, sex, prior concussion history and time since injury as covariates in the statistical analysis. Finally, although the CISS was administered uniformly across sites, variations in verbal versus written administration could potentially affect how patients rate symptom frequency, as previously reported in studies comparing clinician- and self-administered versions of the CISS (Horan et al., 2015; Marusic et al., 2024).

## Conclusion

Adolescents with a history of concussion report significantly higher overall symptom burden on the Convergence Insufficiency Symptom Survey (CISS) compared with visually-normal controls. Higher vision-related and performance-related subscores were associated with the presence of vergence and accommodation deficits, while elevated somatic and performance subscores may reflect increased subjective discomfort and difficulty during visually demanding near tasks such as reading. Concurrent assessment of vergence and accommodation function alongside CISS scores may help identify individuals most likely to benefit from vision rehabilitation aimed at improving vergence and accommodation function and reducing somatic and performance-related symptoms during cognitively demanding near tasks.

## Data Availability

The raw data supporting the conclusions of this article will be made available by the authors, without undue reservation.
