# Supplementary material for: Evaluating post-concussion symptom profiles using the convergence insufficiency symptom survey in a pediatric and adolescent cohort
Source: Front Neurosci. 2026 Mar 25;20:1799528. doi: 10.3389/fnins.2026.1799528 (PMC13056603; doi:10.3389/fnins.2026.1799528)
Supplement: Supplementary file 1 [file Data_Sheet_1.DOCX]

### **Supplemental Methods 1. Additional Eligibility Criteria and Testing Procedures**

**Refractive Correction Requirements**
Participants were required to wear refractive correction if cycloplegic refraction revealed ≥2.00 diopters (D) of hyperopia, ≥1.00 D of myopia, ≥1.00 D of anisometropia, or ≥1.25 D of astigmatism. When correction was indicated, the following standards were applied: for hyperopia, the prescription was reduced symmetrically by no more than 1.50 D in spherical equivalent (SE); for myopia and SE anisometropia, correction was maintained within 1.00 D of full correction; for astigmatism, correction was within 1.00 D of full correction with cylinder axis within 10 degrees for cylinder powers ≥1.00 D.

**Exclusion Criteria**
Participants were excluded if they had a history of amblyopia, strabismus, diplopia, in-office vision therapy, ocular trauma that could affect visual or oculomotor function, or structural abnormalities of the cornea, lens, or central retina. Additional exclusions included presence of constant or intermittent esotropia at distance or near, constant or intermittent exotropia at distance, constant exotropia at near, vertical heterophoria ≥2 prism diopters (Δ) at distance or near, manifest or latent nystagmus, or any neurological or ocular condition known to affect vergence, accommodation, or eye movements. Individuals who were unable to perform study-related vision assessments reliably were also excluded.

**Vergence Testing Procedures**
The near point of convergence (NPC) was assessed using the Near Point Rule (Gulden Ophthalmics, Elkins Park, PA), with a 20/30 vertical column of letters moved from the forehead toward the participant. Participants were instructed to report when the target became double (break point) and when it returned to single vision as the target was moved away (recovery point). Near vergence facility was measured using a hand-held prism flipper (3Δ base-in/12Δ base-out; Gulden Ophthalmics), with participants reporting when the target appeared clear and single for each prism orientation. The number of complete cycles (base-in and base-out) completed within one minute was recorded.

Convergence and divergence fusional amplitudes were assessed at near (40 cm) using a horizontal prism bar ranging from 1 to 40Δ while participants viewed a 20/30 vertical column of letters. The prism demand was increased incrementally, with participants reporting when the image became blurry (blur point), when it doubled (break point), and when it returned to single vision during prism reduction (recovery point). Each of these values was recorded for both convergence and divergence.

**Accommodation Testing Procedures**
Accommodation was evaluated using two monocular tests: amplitude of accommodation (AA) and monocular accommodative facility. AA was measured using the push-up method with a Near Point Rule and a 20/30 vertical column of letters. The target was moved toward the participant until a sustained blur was reported. Monocular accommodative facility was assessed with a ±2.00 D flipper lens and a 20/30 vertical letter column. Participants viewed the target alternately through +2.00 D and –2.00 D lenses and reported when the letters appeared clear for each lens. The number of cycles (one cycle = clarity through both lenses) completed in one minute was documented (Wu *et al.*, 2025).
